# A Proton Beam Therapy System Dedicated to Spot-Scanning Increases Accuracy with Moving Tumors by Real-Time Imaging and Gating and Reduces Equipment Size

**DOI:** 10.1371/journal.pone.0094971

**Published:** 2014-04-18

**Authors:** Shinichi Shimizu, Naoki Miyamoto, Taeko Matsuura, Yusuke Fujii, Masumi Umezawa, Kikuo Umegaki, Kazuo Hiramoto, Hiroki Shirato

**Affiliations:** 1 Department of Radiation Oncology, Hokkaido University Graduate School of Medicine, Sapporo, Hokkaido, Japan; 2 Department of Medical Physics, Hokkaido University Graduate School of Medicine, Sapporo, Hokkaido, Japan; 3 Section of Medical Physics, Hokkaido University Hospital Proton Beam Therapy Center, Sapporo, Hokkaido, Japan; 4 Hitachi, Ltd., Hitachi Research Laboratory, Omika, Ibaraki, Japan; 5 Division of Quantum Science and Engineering, Hokkaido University Graduate School of Engineering, Sapporo, Hokkaido, Japan; 6 Hitachi, Ltd., Research & Development Group, Omika, Ibaraki, Japan; Glasgow University, United Kingdom

## Abstract

**Purpose:**

A proton beam therapy (PBT) system has been designed which dedicates to spot-scanning and has a gating function employing the fluoroscopy-based real-time-imaging of internal fiducial markers near tumors. The dose distribution and treatment time of the newly designed real-time-image gated, spot-scanning proton beam therapy (RGPT) were compared with free-breathing spot-scanning proton beam therapy (FBPT) in a simulation.

**Materials and Methods:**

In-house simulation tools and treatment planning system VQA (Hitachi, Ltd., Japan) were used for estimating the dose distribution and treatment time. Simulations were performed for 48 motion parameters (including 8 respiratory patterns and 6 initial breathing timings) on CT data from two patients, A and B, with hepatocellular carcinoma and with clinical target volumes 14.6 cc and 63.1 cc. The respiratory patterns were derived from the actual trajectory of internal fiducial markers taken in X-ray real-time tumor-tracking radiotherapy (RTRT).

**Results:**

With FBPT, 9/48 motion parameters achieved the criteria of successful delivery for patient A and 0/48 for B. With RGPT 48/48 and 42/48 achieved the criteria. Compared with FBPT, the mean liver dose was smaller with RGPT with statistical significance (p<0.001); it decreased from 27% to 13% and 28% to 23% of the prescribed doses for patients A and B, respectively. The relative lengthening of treatment time to administer 3 Gy (RBE) was estimated to be 1.22 (RGPT/FBPT: 138 s/113 s) and 1.72 (207 s/120 s) for patients A and B, respectively.

**Conclusions:**

This simulation study demonstrated that the RGPT was able to improve the dose distribution markedly for moving tumors without very large treatment time extension. The proton beam therapy system dedicated to spot-scanning with a gating function for real-time imaging increases accuracy with moving tumors and reduces the physical size, and subsequently the cost of the equipment as well as of the building housing the equipment.

## Introduction

Proton beam therapy (PBT) has the potential to create better dose distributions than X-ray therapy in many situations. There are two different types of PBT, passive scattering PBT and spot-scanning PBT [1], [2]. Spot-scanning proton beam therapy (SSPT) is expected to be more suitable to create complex dose distributions and to be safer than the conventional passive scattering method because it reduces neutron contamination [3]. However, there is a larger uncertainty in the dose distribution for tumors in motion due to interplay effects between the time-dependent scanning beam delivery and tumor motion, and this is a disadvantage of the spot-scanning method when compared with passive scattering [4].

The precision of passive scattering proton beam delivery for tumors in motion has been estimated using the motion signals of surface skin markers in Tsunashima et al [5], [6]. It was shown that the synchrotron magnet excitation pattern is an important factor to improve the precision of PBT. However, in clinical situations, it is difficult to achieve dose distributions that closely match the static irradiation, when gating is employed by using the motion of the skin surface, because the internal motion of lung and liver tumors is usually different from the surface motion of the chest wall [7], [8]. This difference may be critical in the case of SSPT. Analysis of the internal motion of fiducial markers near a tumor can be expected to be useful in the evaluation of interplay effects [9]. Also, the cost and size of the SSPT equipment with its advanced technology are further concerns for potential users of SSPT.

We have investigated gated PBT systems dedicated to the spot-scanning method as one solution to improve on these drawbacks. In a previous study, we investigated the dose distribution in a water phantom for a gated SSPT with a gating window of ±2 mm [9]. The three-dimensional (3D) trajectory of a fiducial marker near a lung tumor, which was derived from actual data of internal fiducial markers in real-time tumor-tracking radiotherapy (RTRT), was used in the simulation. That study showed that gated SSPT for a moving target achieved a dose distribution similar to SSPT for a static target. However, it is not obvious whether this result is also satisfied in the human body. There are also concerns about the lengthening of treatment time due to the gating. The 3D motion of tumors is often irregular, involving baseline shifting, and it is also influenced by cardiac as well as respiratory motion, as we have reported elsewhere [7], [10]. If a synchrotron is used without careful attention to these characteristics of tumor motion, on-off signals from internal fiducial markers will change irregularly and sporadically and the treatment time with gated SSPT may become considerably prolonged.

In this study, we investigate the impact of the gated spot-scanning method on proton dose distributions and the lengthening of treatment times in a clinical setting using actual patient computed tomography and recorded data of internal tumor motion.

## Materials and Methods

**Figure 1:**
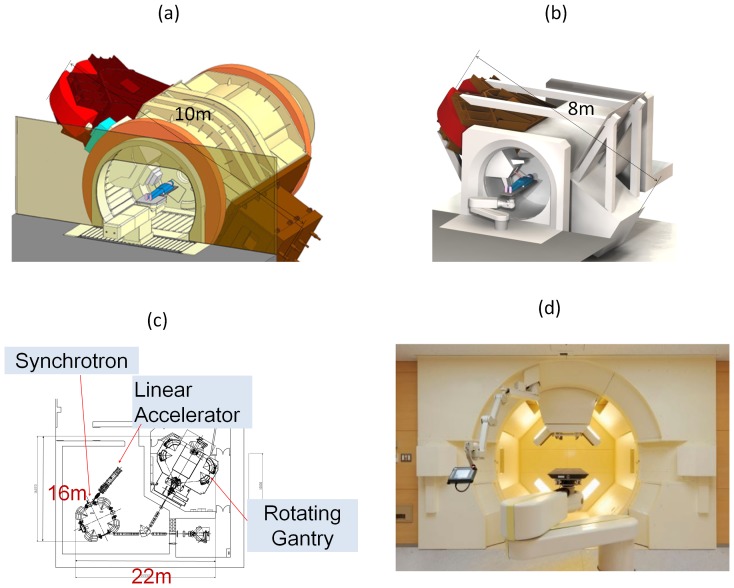
RGPT system at Hokkaido University. (a) The gantry of the passive scattering PBT system. (b) The gantry of the RGPT system. (c) The footprint for one gantry and one fixed beam system with a linear accelerator and synchrotron, and (d) The actual RGPT system installed.

We have been developing an RGPT system since 2009 (Figure 1). We have found that the cost for the system and related buildings are decreased, not increased, by dedicating to SSPT. This is possible because we can avoid the scattering components in the nozzle and reduce the size of the equipment and the overall footprint. The radius of the gantry can be shortened from 5 m to 4 m and the weight reduced from 160 tons to 100 tons. Since there is no energy loss with the absent of scattering components, the maximum energy to treat tumors at 30 cm depth in a human body can be reduced from 250 MeV to 220 MeV. The efficiency of the proton beam increases from about 20% to about 100%, and thus the accelerated proton current can be reduced from 10 nC to 2 nC. Consequently, the size of the synchrotron becomes more compact than with passive scattering systems, its circumference being reduced from 23 m to 18 m. A 35% reduction, from 546 m^2^ to 352 m^2^, was achieved in the footprint for a system with one gantry and one fixed beam with the capacity to expand to a system with multiple gantries. The total cost was reduced accordingly. Acceptance tests are in progress, and clinical use of the system is to be started from March 2014.

**Figure 2:**
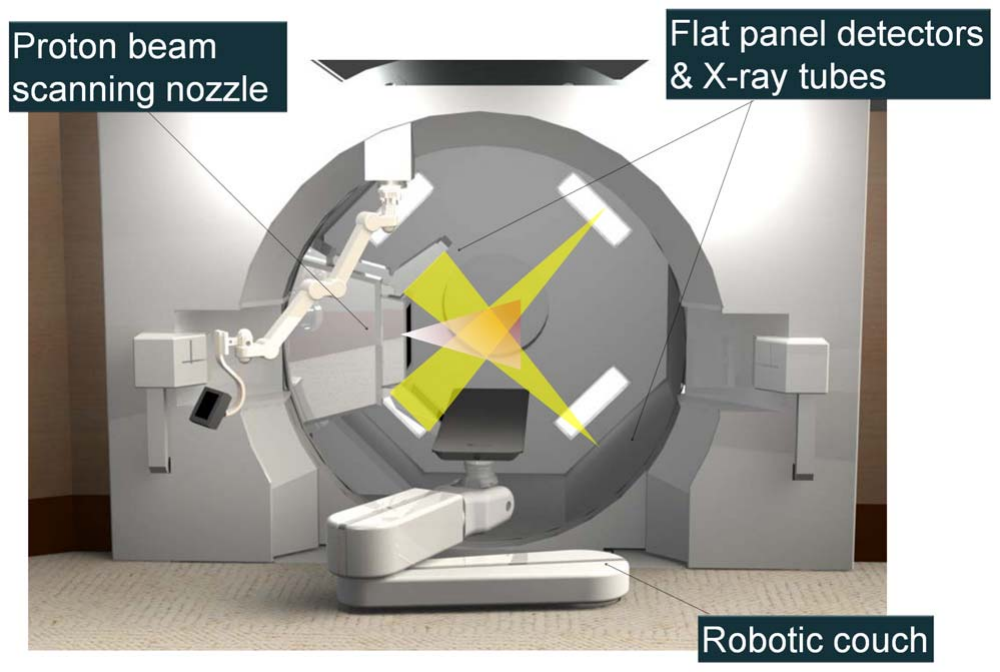
Design of a spot-scanning proton beam therapy-dedicated system with X-ray fluoroscopy. Two orthogonal sets of X-ray fluoroscopic generators and flat panels can be mounted in the gantry.

As an SSPT-dedicated system does not require compensators and collimators, in principle, we are able to install two orthogonal sets of X-ray fluoroscopes in the gantry (Figure 2). The X-ray images can be acquired simultaneously with proton beam irradiation, and their fields of view will not be narrowed by these field-shaping devices. Cone-beam computed tomography, which is useful to reduce inter-fractional set-up errors, can be created by rotating the gantry using the X-ray fluoroscope [11]. By using simultaneous orthogonal fluoroscopic real-time images, fiducial markers near the tumor can be detected every 0.033 s with a delay of about 0.05 s and with an accuracy of 1 mm during the delivery of the SSPT, as in RTRT with X-ray therapy [12]. The tumor is irradiated only when its location corresponds to the planned position within an accuracy of ±1–2 mm by gating the proton beam using the real-time-images. This function can reduce random errors, as is well known from the use of RTRT in X-ray therapy. In this study, the SSPT system is termed a real-time-image gated proton beam therapy (RGPT) since the proton beam is gated with the real-time-images of fiducial markers in the patient being treated.

**Figure 3:**
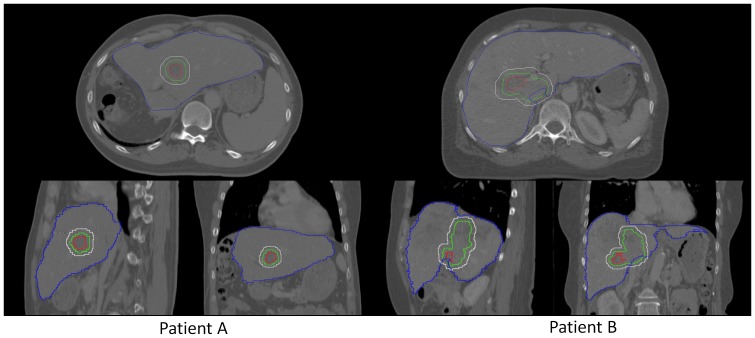
CT images and target delineations. Computed tomography for the patients with hepatocellular carcinoma included in this study. Gross tumor volumes (red), clinical target volumes (green), liver (blue), and planning target volumes for the RGPT (white) for patient A (left) and patient B (right).

**Figure 4:**
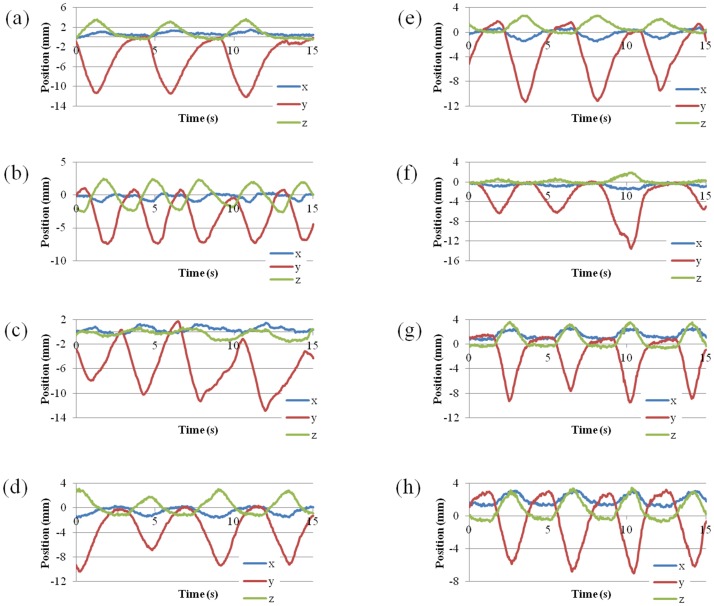
Tumor motions obtained with RTRT system. Eight patterns of tumor motion derived from actual data of internal fiducial markers near hepatocellular carcinomas in the X-ray RTRT of 8 patients.

The study investigates the efficacy of RGPT, comparing it to non-gated free-breathing SSPT (FBPT). CT data sets of two patients with hepatic carcinomas were used for the comparison, and CT images were taken at the end of the exhale phase with a 2.5-mm slice interval using a 64 multi-detector CT scan. The total liver volumes were 1105 cc and 1688 cc, respectively (Figure 3). The two tumors had clinical target volumes (CTV) of 14.6 cc and 63.1 cc, respectively. We assumed administration of 3.0 Gy (RBE (relative biological effectiveness)  = 1.1) at the isocenter of the CTV. We further selected 8 representative patterns of tumor motion derived from the actual data of internal fiducial markers near hepatocellular carcinomas in X-ray RTRT (Figure 4). The amplitude and data acquisition time for each fiducial marker are shown in Table 1. As we have reported elsewhere [8], the 3D coordinates of the internal fiducial markers were detected and recorded every 0.033 s using two sets of fluoroscopes in the X-ray RTRT. The shift of the marker positions from the planned (original) positions was assumed to represent the shift of the tumor in this study. For simplicity, we have not included the deformable nature of patient body in the simulation, which would lead to the additional range changes in the beam path. The 8 patterns of tumor motion were applied to the 2 hepatic tumors in the following simulation, and for each pattern, 6 different initial breathing timings were considered at 1 s intervals.

**Table 1:** x, y, and z components of the amplitude of movement of a fiducial marker near the tumor, and data acquisition time, in 8 patients with hepatocellular carcinomas.

Motion ID	x(LR) (mm)	y(SI) (mm)	z(AP) (mm)	Data acquisition time (s)
a	2	15.5	5.7	128
b	2.1	10.7	6.1	133
c	2.4	18	2.8	104
d	2.7	20	7.7	160
e	2.8	17.6	3.9	155
f	1.7	14	2.6	85
g	3.1	12.1	4.8	77
h	2.4	10.2	4.3	47

Data were taken by a X-ray real-time tumor-tracking radiotherapy (RTRT) system.

The static plans were generated in a treatment planning system, VQA (Hitachi, Ltd., Japan). Parameters used in the treatment planning for each patient are shown in Table 2. Using the beam scanning sequence of a PROBEAT series proton accelerator (Hitachi, Ltd., Japan), each beam position was translocated by the values of the tumor motion at the moment of the spot irradiation using in-house simulation tools. Then, the dose distributions were calculated using the actual spot positions. In the treatment planning, the planning target volume (PTV) was drawn as the geometric expansion of CTV as in the photon planning. For RGPT, PTV was defined as PTV_rg_  =  CTV + an internal margin (3 mm) + a residual margin (2 mm) and for FBPT, PTV_fb_  =  CTV + an internal margin (5 mm LR, 5 mm AP, 10 mm SI) + a residual margin (2 mm). Here, the internal margin is added to compensate for the target motion, which mainly results from respiration and cardiac motion, while the residual margin is added to compensate for the various set-up uncertainties.

**Table 2:** Parameters used in the treatment planning.

Patient ID	A	B
Gantry angle	0°	270°
PTV (cm3)	38.7(RGPT), 123.3(FBPT)	134.3(RGPT), 204.2(FBPT)
Energy (MeV)	89–123(RGPT), 82–128(FBPT)	97–152(RGPT), 93–155(FBPT)
Number of layers	29(RGPT), 41(FBPT)	36(RGPT), 41(FBPT)
Spot interval (mm)	6	6
Max. MU/spot	0.04	0.04
Number of spots	2015(RGPT), 4638(FBPT)	4025(RGPT), 5314(FBPT)

**Figure 5:**
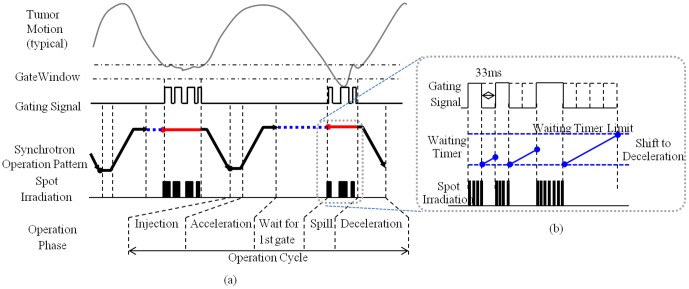
Diagram of the (a) synchrotron operation and (b) beam waiting function. The operation cycle of the synchrotron varies approximately from 2 to 7

The synchrotron operation pattern and beam waiting function are schematically drawn in Fig. 5. The synchrotron operation in gating mode has the operation phases of injection, acceleration, wait for first gate, extraction, and deceleration. The operation cycle of the synchrotron varies approximately from 2 to 7 s. The acceleration and deceleration both require about 1 s. The flat top length, which consists of wait for first gate and extraction time, has a maximum of 5 s. Beam waiting function was installed to this system in order to improve irradiation efficiency. This function enables to multiple gate irradiations per synchrotron operation cycle. In this function, waiting timer limit 200 ms has been applied from the view point of stability of circulating proton in the synchrotron and beam irradiation efficiency [13].

In this simulation, the maximum flat top and extraction time were set to 5 s and 4.4 s, respectively. The beam delivery started from the deepest layer to the shallowest layer and each iso-energy layer was individually rescanned. From the viewpoint of safety, the maximum deliverable dose per spot is limited to 0.04 in the monitor unit (MU) and the delivered dose is checked spot by spot, where the definition of MU is the same as that described in Gillin et al [14]. When the planned MU for a spot exceeds the pre-defined value under the limit of 0.04, the spot is revisited. We set the pre-defined value to 0.04, which indicates the minimum number of repeated deliveries is performed. Note that although a larger number of rescanning is expected to wash out the hot and cold spots that may occur as a result of the interplay effect, it should also lengthen the irradiation time. The scanning speed, the beam current, and the gating window were set to 10 m/s, 7.5 MU/s, and ±2 mm, respectively. The latency of the system is defined as the duration between the generation of the pulsed x-ray beam of the fluoroscope and the resultant proton beam-on/off, including the image acquisition/procession; it was set to 66 ms.

The mean of mean live dose (MLD) among 8 patterns of tumor motion in FBPT was compared with the mean of MLD in RGPT using paired t-test in each patient. The Ansari-Bradley test was used for comparisons between RGPT and FBPT on the maximum and minimum doses in the CTV.

This study has been approved by the institutional review board of Hokkaido University Hospital (011-0124). The requirement for written consent was waived by our institutional board according to the Ethical Guidelines for Clinical Studies of the Japanese Ministry of Health, Labour and Welfare.

## Results

The total irradiation time in RGPT and FBPT for each patient was estimated to irradiate 3.0 Gy (RBE) at the isocenter (Table 3). In RGPT, the mean treatment times for PTV_rg_ of patient A and patient B were 138 (96–185) s and 207 (126–314) s, respectively, where the range of values is shown in parentheses. If we irradiate the PTV_rg_ to a static target, the treatment time is estimated to be 74 s and 101 s, respectively. Thus, the physical ratios of lengthening of the treatment time due to the gating were 1.87 (138/74) and 2.05 (207/101) for patients A and B, respectively. However, the PTV_rg_ does not include internal margins in the free-breathing mode and is too small for actual irradiation with free-breathing. Treatment times for PTV_fb_, the PTV including the internal margin in free-breathing, were 113 s and 120 s for patients A and B, respectively. Therefore, the clinical ratios of lengthening of the treatment time were estimated to be 1.22 (138/113) and 1.72 (207/120), respectively. The minimum treatment time with RGPT for PTV_rg_ (96s) was shorter than the treatment time with FBPT for PTV_fb_ (113s) due to the differences in the volumes of PTV_rg_ and PTV_fb_ (Table 3).

**Table 3:** Treatment times for FBPT and RGPT.

Patient ID		A	B
FBRT for PTVfb		113	120
RGPT for PTVrg	static (s)*	74	101
	mean (s)	138	207
	max (s)	185	314
	min (s)	96	126

Mean, maximum, and minimum values are those of 8 respiratory patterns and 6 initial timings of motion data.

*: Treatment time required for static irradiation for PTV_rg_, which is smaller than PTV_fb_.

**Figure 6:**
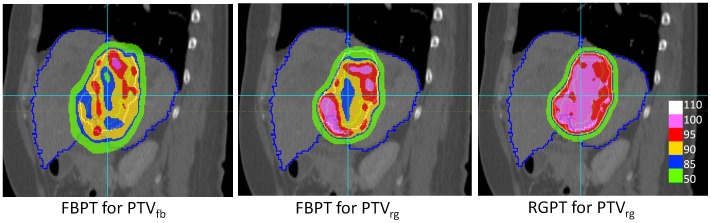
Comparison of dose distribution between FBPT and RGPT. Images of the dose distributions with FBPT for PTV_fb_(left), FBPT for PTV_rg_ (center), and RGPT for PTV_rg_ (right) for the CT images of patient B with tumor motion ID of b.

Table 4 details the maximum and the minimum doses in the CTV relative to the prescribed dose at the isocenter of the CTV for the combination of the 2 targets, 8 respiratory patterns, and 6 initial breathing timings. Based on the recommended dose homogeneity in ICRU (1999) [15], we considered that the dose was successfully delivered to the CTV if the dose in CTV was within the range between 95% and 107%. In the FBPT, 9 of 48 (9/48) and 0/48 motion parameters (including respiratory patterns and initial breathing timings) achieved the criteria for successful delivery, whereas in the RGPT, 48/48 and 42/48 achieved the criteria for patients A and B, respectively (Figure 6). The improvement in success rate was more pronounced in patient B, with a larger PTV. As shown in Table 4, the maximum to minimum range of 6 initial breathing timings was apparent in FBPT but was quite small in RGPT. The Ansari-Bradley test were applied and showed that RGPT reduced the variances in either maximum or minimum doses in 7 of 8 respiratory patterns in patient A and 6 of 8 in patient B with statistical significance (p<0.05) (Table 4). It reduced the variances in both maximum and minimum doses compared with the FBPT in 6 of 8 respiratory patterns in patient A and 4 of 8 respiratory patters in patient B with statistical significance (p<0.05). These facts suggest that the influence of the initial breathing timing on the dose homogeneity can be reduced in RGPT compared with free breathing in the majority of situations. The mean liver dose for the liver volume minus the gross tumor volume (MLD) was also estimated and is shown in Table 4. Compared to FBPT, MLD was reduced in RGPT with statistical significance, from 0.27 to 0.13 (p<0.001) and from 0.28 to 0.22–0.23 (p<0.001) for patients A and B, respectively. The reduction rates of MLD were 51.9% and 17.9% for patient A and patient B, respectively. There was no apparent difference in the ratio of reduction in MLD between the 8 patterns of tumor motion in the 2 patients (Table 4).

**Table 4:** Maximum and minimum doses in CTV scaled by the prescribed dose, and mean liver doses in FBPT and in RGPT, averaged over 6 initial timings of motion data.

**FBPT**						
Motion ID	Patient A			Patient B		
	Max	Min	MLD	Max	Min	MLD
static	1.01	0.98	0.27	1.02	0.98	0.28
a	1.11(1.20–1.06)	0.93(0.96–0.81)	0.27	1.18(1.26–1.09)	0.78(0.83–0.72)	0.28
b	1.05(1.11–1.01)	0.94(0.98–0.90)	0.27	1.11(1.14–1.08)	0.86(0.91–0.82)	0.28
c	1.08(1.15–1.03)	0.95(0.98–0.89)	0.27	1.13(1.26–1.08)	0.79(0.87–0.70)	0.28
d	1.06(1.10–1.03)	0.94(0.98–0.91)	0.27	1.19(1.26–1.11)	0.85(0.91–0.76)	0.28
e	1.07(1.11–1.03)	0.92(0.98–0.89)	0.27	1.15(1.20–1.11)	0.81(0.88–0.75)	0.28
f	1.05(1.07–1.00)	0.92(0.98–0.80)	0.27	1.09(1.10–1.07)	0.90(0.97–0.79)	0.28
g	1.05(1.11–1.03)	0.95(0.98–0.93)	0.27	1.16(1.29–1.09)	0.85(0.91–0.80)	0.28
h	1.05(1.11–1.01)	0.94(0.99–0.89)	0.27	1.19(1.29–1.06)	0.87(0.93–0.76)	0.28
						
**RGPT**						
Motion ID	Patient A			Patient B		
	Max	Min	MLD	Max	Min	MLD
static	1.02	0.99	0.13	1.03	0.98	0.23
a	1.02(1.03–1.02)	0.98(0.98–0.98)	0.13	1.03(1.03–1.03)	0.97(0.97–0.96)	0.22
b	1.02(1.03–1.02)	0.98(0.99–0.98)	0.13	1.05(1.05–1.03)	0.96(0.97–0.95)	0.22
c	1.03(1.04–1.02)	0.98(0.98–0.96)	0.13	1.03(1.03–1.03)	0.96(0.96–0.96)	0.23
d	1.02(1.02–1.02)	0.98(0.99–0.98)	0.13	1.05(1.06–1.04)	0.97(0.98–0.96)	0.22
e	1.03(1.03–1.03)	0.97(0.97–0.96)	0.13	1.06(1.06–1.05)	0.95(0.96–0.94)	0.23
f	1.02(1.02–1.01)	0.98(0.99–0.97)	0.13	1.04(1.04–1.04)	0.98(0.98–0.98)	0.22
g	1.03(1.03–1.03)	0.98(0.99–0.97)	0.13	1.05(1.08–1.03)	0.96(0.98–0.96)	0.23
h	1.03(1.03–1.02)	0.98(0.98–0.98)	0.13	1.03(1.04–1.02)	0.97(0.97–0.96)	0.23

The range of values are shown in brackets.

## Discussion

The present study found that the dose distribution with hepatic carcinomas can be improved in MLD by using RGPT with statistical significance, when compared with FBPT, based on a comparison using actual data sets of the internal motion of fiducial markers. It is notable that RGPT was able to provide 95–107% coverage of CTV even with the minimum number of repaintings. In addition to the better dose distribution in CTV for RGPT, the MLD can be reduced by about 50% in patient A, who had a small tumor, and by about 20% in patient B, who had a large tumor. We also found that the reduction rate of MLD did not depend on the patterns of tumor motion but on the size of the target. The large reduction rate for patient A with the small tumor suggests that RGPT would be suitable for patients with poor hepatic function and with small tumors, and it is worth noting in this context that patients with small tumors could be adequately treated with X-ray stereotactic radiotherapy in our previous study [16].

Another important finding here is that the treatment times were not prominently lengthened when using RGPT. From our clinical experience through X-ray treatment, the lengthening ratios of 1.22 and 1.72 that we observed in this study would be manageable. With respect to the evaluated treatment time, the additional dose due to real-time imaging is estimated to be less than 50 mGy, assuming the typical imaging conditions used in a conventional real-time imaging system. An accumulated imaging dose would be clinically acceptable. It must be noted that reductions in the PTV can greatly shorten the treatment time in SSPT. Adding to this difference due to the size of the target volume, a further developed gating function will reduce the estimated treatment time [13]. The new function enables multiple gated irradiations per synchrotron cycle, to improve the irradiation efficiency and reduce treatment times.

A shortcoming of this study is the use of a rigid motion model in the simulation; the scanning beam was assumed to change position relative to the static CTV in the human body. The dose distribution would change if the changes in the water-equivalent path length along the beam path were correctly included in the simulation. The number of fields also can affect the results. A precise analysis of these effects should be performed using 4D computed tomography or 4D magnetic resonance images, and the verification by actual measurements has to be done in future [17]. Also, it is important to note that to confirm this new system delivers the treatment as expected, we have to examine patient outcomes over time.

The effect of gold markers on the dose distribution has been known to be a potential hazard in particle therapy. Since SSPT does not require compensators and collimators, SSPT is suitable for multiple beam treatment. In a previous study, the effect of gold markers was found to be biologically negligible if multiple beams were used to irradiate the target [18].

In conclusion, spot-scanning proton beam therapy can improve dose distribution with real-time imaging and gating. There is no serious lengthening of treatment times with the real-time-image gating because of the smaller internal margin and new developments in gating functions. The results and considerations here allow the conclusion that a proton beam therapy system dedicated to spot-scanning and incorporating the capability for real-time imaging and gating can reduce the total size and cost of the equipment and facilities.
